# Patients’ ideas, expectations and experience with self operated endovaginal telemonitoring: a prospective pilot study

**Published:** 2017-09

**Authors:** S Verdonckt, J Gerris

**Affiliations:** Women’s Clinic, University Hospital Ghent, De Pintelaan 185, 9000 Ghent, Belgium

**Keywords:** ART, home sonography, patient reported outcomes, telemedicine, patient empowerment

## Abstract

**Objective:**

To examine advantages and disadvantages as perceived by patients and their partners using home sonography for monitoring ovarian stimulation prior to artificial fertility treatment.

**Method:**

We interviewed 25 patients and their partners and took 44 online questionnaires. All interviews were written out and the transcripts were coded, based on words patients used to describe their experience. The query consisted mostly of statements, of which the participant had to ascertain whether or not they agreed (1= I absolutely don’t agree and 5 = I absolutely agree). The median and mean of agreement scores was calculated.

**Results:**

The time saving and practical aspect of self-operated endo-vaginal tele-monitoring (SOET) was the most important argument to choose SOET. In addition, the following aspects were considered advantages: more autonomy, no need for leave from work, a better doctor-patient relationship and sometimes more involvement of the partner. The most important disadvantage is a sense of initial insecurity couples experience during the first ultrasound. Almost all couples experience this, but they accept it as part of the process.

**Conclusion:**

Using SOET was a pleasant experience for all couples. All patients and partners had positive expectations about SOET. The initial insecurity can be minimized, by improving teaching measures. It would be positive if a legal framework is set up allowing reimbursement of home sonography.

## Introduction

The focus of this article is patients’ and their partners’ view on self-operated endo-vaginal telemonitoring (SOET). In our current climate of health care, where both patient-centered care and saving time and money are important issues, telemedicine is becoming increasingly important, for it stands for more out-hospital care. Telemedicine is an umbrella concept referring to all systems that substitute the classical means of communication by electronic communication. (Bashshur et al., 2011). It is not bound to one discipline, but can be used as a tool to ease the daily routine in several medical applications. SOET can be classified as an asynchronous m-health application, which means that it uses indirect communication and it is a mobile application. There are a few obstacles that stand in the way of a broad use of the SOET system.

The most important one in Belgium is the lack of legislation about telemedicine. In addition, there is no reimbursement for this technology, which means that the patients bear all the costs of telemedicine. Politicians in charge of social affairs, want to tackle this problem, but for now no specific measures have yet been taken.

**Figure 1:**
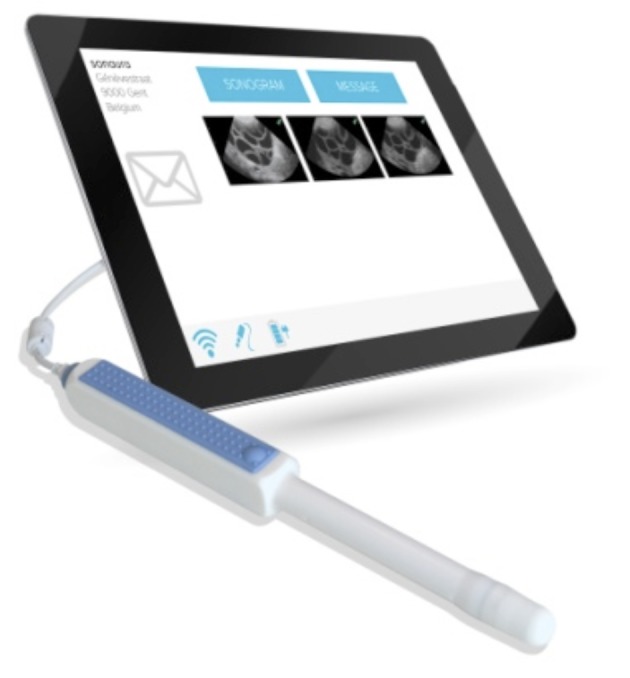
— ‘The SOET device: a tablet and a USB-connected probe’

During an artificial reproduction treatment (ART), patients have to be monitored by serial endo-vaginal ultrasounds. For the patients living far away, this requires a lot of organisation. SOET was developed to solve this specific problem. It is a cloud-based application that allows patients to perform sonograms at home and send them to their doctor using specially designed, safe software. Once a patient decides to use SOET, she receives a case with a tablet, a usb connectable echoprobe and a personal password (Fig. 1). She then is given a comprehensive explanation and a demonstration of the application. The patient makes the ultrasound and sends it to the physician, whenever it suits her and from wherever wifi is available. The goal is to record the ovaries for 30 seconds each and the uterus for 15 seconds. The doctor receives a notification on his mobile phone that video recordings have arrived, watches and interprets the images on his PC and sends clear instructions to the patient. If there are any questions, the patient can send a message through the application. If the video images are not clearly interpretable after a maximum of 12 days, which occurs very rarely (about 1-2% of all attempts), patients are called to the hospital. After the final ultrasound, clear instructions are sent for the administration of hCG (Gerris et al., 2016).

Today, four trials have been conducted on the subject of SOET (Gerris et al., 2009, 2014, 2016; Pereira et al., 2016). From these studies it can be upheld that SOET is an equal alternative to incentre ultrasound in terms of number and quality of follicles, conception, number of embryos and pregnancy chances. Patients reported a higher contentedness, a higher feeling of empowerment, less stress, less productivity loss and lower transportation costs. Almost all attempts could be completed without any hospital visit between the demonstration ultrasound and oocyte retrieval.

Although Gerris et al. (Gerris et al., 2009; 2014; 2016) questioned patients already about their ideas and expectations of SOET before the concept was worked out clinically, the present study was in fact the first systematic study to examine, not only the patients’, but also the partners’ opinion about SOET at a time where several hundreds of attempts have been performed and experience could be gained at the physicians’ side as well.

## Methods

This investigation is a qualitative and semi- quantitative study, for which we used online queries and in-depth interviews to learn about the experience with and the ideas and expectations about the SOET-application. We chose questionnaires to get a brief and clear idea about the participants’ experience and performed interviews to get a more extensive view on their experience or expectation. This was approved by the ethical committee with the registration number B670201525902. The study was conducted between September 2015 and June 2016.

During routine consultations, patients and their partners were asked if they wanted to participate in the study. They were sent to a separate office for a broader explanation of the study and if they decided to take part, both patient and partner signed an informed consent form. The query consisted mostly of statements, of which the participant had to say whether or not they agreed (1= I absolutely don’t agree and 5 = I absolutely agree). Questions about fertility treatments, about using SOET, about the possible advantages of SOET and about performing the ultrasounds and the associated emotions. A link was sent to the email-address that the couple left behind the day of recruitment. The results of the query were automatically transported to Microsoft Excel and analysed. Patients and partners were analysed separately and were divided into three categories: those who had not yet decided if they would start using SOET or the classical follow- up, those who were going to start SOET and those who had already used and experienced SOET. The median and mean of agreement was calculated, as we visioned agreement more as a continuous variable than a categorical. Because of the nature of this semi-quantitative and qualitative study, we decided right from the beginning not to perform extensive statistical analysis on the results, but to describe the participants’ experience.

If the patient agreed with an interview, it was performed right after the explanation about the query. With their consent, the interview was taped and afterwards written out. The interviews were divided in the same categories as the queries for the analysis. The transcript was read and the relevant parts were coded and recoded until there were a few larger categories left. Codes were allocated based on words patients used to describe their experience. The amount of times a certain code occurred, was counted and taken into account.

## Results

During recruiting, 22 patients and 22 partners were asked to participate in the study. 14/22 patients and 7/22 partners filled in the query and 13/22 patients and 12/22 partners took the interview. Table I summarizes the demographics of the population and their history of ART.

**Table I:** Demographical and medical information about the participants

		Patients (n = 14)	Partners (n = 7)
Mean age		33.7	42.4
Nationality			
	Belgian	0	0
	Dutch	14	7
Country of residency			
	Belgium	0	0
	The Netherlands	14	7
Marital status			
	married/living together	14	7
	single	0	0
	other	0	0
Mean distance back and forth (in km) from residency to the clinic		332	325
Mean time from residency to the clinic (in min.)		255	263
cost estimate for transportation (in euro)		29	33
Do you mostly consult together with your partner?			
	yes	14	7
	no	0	0
Mean amount of ART-treatments		4	5
Mean amount of years undergoing ART-treatments		4	4
Which type of treatment are you/your partner currently undergoing?			
	IVF	0	0
	ICSI	14	7
Are you currently IN a SOET-cycle (at least 1 sonography sent) or have you done a SOET-cycle in the past?			
	yes	9	5
	no	1	1
	starting	4	1
Have you been pregnant because of an ART-treatment?			
	yes	8	-
	no	6	-

### Questionnaires

For the results, only three groups were analysed: patients who started SOET (group A) and patients (group B) and partners (group C) with experience in SOET. The three other groups - partners starting SOET and patients and partners still in the decision process – did not contain enough participants to analyse. Results of the first part of the questionnaire showed that all participants found the treatment easier to organize in their daily life. This was mostly explained by the lesser hospital visits (6/9 group B, 4/5 group C and 4/4 group A). More than half of the experienced patients and all of the starters preferred to make their own ultrasounds, rather than to make a time-consuming trip to the hospital. The fact that the participants can manipulate their own/their partner’s private parts, doesn’t seem to be an important benefit (no participants agreed on this statement).The majority of patients (3/4 group A, 5/9 group B) are pleased that they have more autonomy with SOET. 3 out of 5 partners thought it was nice to actively take part in the treatment. In the second part of the query, participants were asked about the potential benefits of SOET. The most important advantage for all patients and partners are the fewer hospital visits and the (possible) time saving aspect of this (4/4 group A, 8/9 group B, 5/5 group C). Patients experience less stress, because of the organizational simplification (4/4 group A, 8/9 group B). Not having to take time off of work is an important benefit, according the respondents (4/4 group A, 5/9 group B, 5/5 group C), as their privacy is respected this way. Finally, saving gas money seems to be less important, mostly for the patients (1/4 group A, 1/9 group B, 2/5 group C).

In the third part of the query, respondents were asked about the use of SOET. For the vast majority, the demonstration in the first consultation was clear (3/4 group A, 9/9 group B, 5/5 group C). This gave 8/9 experienced patients and all partners the confidence they needed to make their own ultrasound. On the Sonaura® website, instructional videos and written instructions are to be found, which were clear for 8/9 patients and 4/5 partners. All patients and partners were able to make their ultrasound at the right time. All participants were very enthusiastic about the communication with the doctor and found it sufficient to communicate and receive instructions solely through the SOET- application. They felt like they could create a steady trustworthy relationship with their physician. 7/9 patients and all partners agreed that the software and the usb-ultrasound probe was easily manageable. Half of the patients (5/9) and partners (3/5) felt secure about the quality of their sent images, but 8/9 patients found the ultrasounds to become easier as their cycle continued. All patients wanted their partners to be very involved in the treatment, as do 4/5 partners. 5/9 patients thought their partner was more involved with the SOET-cycle and 3/5 partners felt more involved. Finally, 7/9 patients and 3/5 partners felt less stress during the ovarian stimulation phase of the ART in comparison with the classical treatment.

At the end of the query, we asked if they would use SOET again, if necessary. All patients and partners gave a positive answer. When asked if they would recommend SOET to befriended couples 7/9 patients and 3/5 partners said they would. The others would consider the personality of the couple first.

### Interviews

In addition to the questionnaires, we interviewed twenty-five participants about their experience with or expectations about SOET. The results largely corresponded to the results of the questionnaires. When participants were asked about their general opinion of SOET, all were positive. All of the experienced patients as well as starters saw the (possible) advantages of the system. Some of the participants were anxious to make the first ultrasound by themselves, seen as this is a totally new experience.

Despite this possible disadvantage, the organizational convenience of SOET convinced most patients to choose for SOET. They experienced more emotional stability and less burden, saved a lot of time and had less absence from work. The time saving aspect dominated most participants’ reasons to choose SOET. The fact that the sonographies can be made where and when it suits the patient, empowers the patient and stimulates her feeling of autonomy. The ultrasounds are made in familiar surroundings in a serene atmosphere, without commuting in busy traffic, which lowers the stress levels for both patients and partners.

In the beginning of the SOET-cycle, most patients and partners do feel insecure about their capacity to make good ultrasounds. This initial insecurity was experienced by almost all of the couples, mostly at the start of the first cycle. In the majority of cases, this insecurity disappears soon, certainly when they receive a positive, encouraging message from their doctor. Most of the participants had multiple ART attempts in the past, so they had some experience with ultrasound. This made the decision for SOET easier and raised their confidence. Although all couples experience this insecurity, they accept it as part of the process.

Another aspect of the application that could lead to some additional stress is the technological side of the story. Some patients experienced some minor technical problems (internet connection, saving videos), but the item that was mostly mentioned, is the difference in resolution between their screen and the ultrasound machine at the hospital. Couples are used to see their ultrasounds on high- resolution machines in the hospital in comparison with which the images on the tablet are of lower quality. Some couples suggested using Sonaura for the demonstration sonography, to get used to the device and the resolution. Most couples thought it was normal that the first time is clumsy. One couple suggested that some practical, hands-on tips would be nice before performing the first ultrasound.

As mentioned before, in the results of the query, patients and partners felt like they could build a good trusting relationship with their doctor, despite the lack of face-to-face contact. Most couples felt they had a better relationship with their doctor than in previous treatments. They gave two possible explanations for this: the fact that they were followed by one doctor only and the way of communicating. The fact that their doctor wrote them not only short, medical messages, but also engaged in some small- talk was very much appreciated.

Finally we asked about the involvement of the partner in the treatment. The answers to this question were divided. The partners who made the ultrasound themselves felt a much higher involvement, but the other partners did not notice a striking difference.

## Discussion

We can state that the main experience of SOET is positive, for all participants. The fact that the couples can make their ultrasound at home is, for most couples, the decisive reason to choose SOET. Both patients and partners feel less organizational stress and more emotional stability during the treatment. Patients do not have to take leave at work and can keep their fertility treatment private. This need for privacy is an important issue. Therefore, the data transmission of SOET currently meets all technical requirements and privacy-standards of bank-data. An important topic that arises from both parts of the research is the initial insecurity. The fear of not being able to make good ultrasounds, can be a reason not to choose SOET. Experienced couples confirm that the first ultrasounds make for exciting and stressful moments, but as the cycle continued, the stress lessened and the ultrasounds became easier to make. This has two possible explanations: the follicles grow and become increasingly easy to find and there is a natural learning curve for sonography (Gerris et al., 2009). In the following, we list a few suggestions for improvement in that area. Today, the demonstration ultrasound is done with a high quality ultrasound, which is interesting for the patient, because it makes a clear view. However, this is somewhat misleading, because the resolution of the SOET device is a lot lower. To lower the threshold for the first ultrasound, it might be a good idea to make the demonstration ultrasound with the SOET device. Or one can go even further and let the patients make their own first ultrasound at the consultation. Another possibility is to let patients make their first sonography at home, but in direct contact with the doctor. In these situations, the patient or partner can try their first ultrasound in a controlled and ideal situation. Another way to boost patients’ confidence, is to start a patient community for SOET-users. In concrete terms, an online forum could be established, where partners and patients can exchange practical tips and experiences. This can be valuable for starting patients as for experienced patients.

The most surprising result of the study was that we found that the doctor-patient relationship is found to be better and more intense. Despite the asynchronous communication, the couples could build a good relationship with their doctor. With the SOET-application, the follow-up was done by one and the same doctor, this in contrast to the classical treatment. In addition, the way of communication was also very important. Most doctors are not used to communicate through messages. As telemedicine is booming, there should be considered to include a course about electronical communication in the education of future doctors.

The findings of this study are comparable to a bigger study of Agha et al. (2009), who researched the patient satisfaction with telemedicine.

For patients as for partners, their involvement in the fertility treatment is very important. With SOET, there is the possibility for the partners to feel much more involved, by performing the ultrasounds themselves. In about half of the cases, the partner made the images. We found that the partners, who made the ultrasounds, felt more involved because of SOET, the others didn’t notice a great difference in involvement.

### Implementation

Although all previous results are promising, there are a few obstacles for the implementation of SOET. The most important one is the lack of legal framework and reimbursement. The current ministry of health wants to invest time and money in tele-health, but no concrete measures were taken yet. Moreover, there is a chance that other, bigger disciplines (like diabetes-care) will be granted priority. This legislation is very much nationally determined and in different countries, this might not be an issue. The implementation of SOET in the daily practice, asks for some adjustments. The first consultation will take longer, the doctor has to find time to measure the ultrasound and in bigger hospitals, echographists will have less work. These disruptive changes are not easy and ask a mentality switch in the clinic. When a part of the patients would ask SOET though, this could lead to a less busy practice and more time for the patients at the time of consultation. To measure the time-saving aspect of SOET, we should establish a comparative study between a doctor following patients under the classical treatment and a doctor following a mixed group of SOET and not-SOET patients.

Finally, inclusion or exclusion factors for SOET should be considered. In the research of Pereira et al. some parameters were suggested. They found that BMI has a predictive value for the quality of the images. However, they only studied women who used SOET just once, not for a whole stimulation period. (Pereira et al., 2016)This aspect of SOET needs further investigation. Response in previous ART can also be considered as an exclusion factor. Patients that were called back to the hospital, were mostly extremely poor responders.. If these poor responders are identified at the start, this can save them a possible disappointment. OHSS in previous ART is considered a risk factor, but not an exclusion criterion. Finally, previous experience with ART’s can be considered a relative inclusion criterion. To define the objective criteria, more research is needed.

## Conclusion

Using SOET was a pleasant experience for all couples and all starting patients and partners had positive expectations about SOET. The initial insecurity can still improve, if certain limited teaching measures are taken. It can be valuable to study the experience of the partner. Although patients do not report that the cost of SOET is too high, it would be positive if a legal framework is set up. Follow-up ultrasounds make out 7% of the total cost of an ICSI-treatment and are currently charged with the fee for service principle. All other aspects of the treatment are payed for by a xed amount or a particular health insurance identification number. We recommended to cover the ultrasound follow- up with a fixed amount so that the whole fertility treatment is covered by one global amount.. This way, couples can choose between a classical treatment or SOET, without additional cost.

## Declaration

The author report no financial or commercial conflicts of interest.
